# Sclerosing Epithelioid Fibrosarcoma of the Parietal Bone and Adjacent Meninges in an Adolescent: A Case Report

**DOI:** 10.4021/wjon742w

**Published:** 2014-01-16

**Authors:** Sasikumar Kilaikode, Sandeepkumar Kuril, Aziza Sedrak, Swayam Sadanandan

**Affiliations:** a Department of Pediatrics, The Brooklyn Hospital Center, 121 Dekalb Ave, Brooklyn, NY 11201, USA; b Division of Pediatric Hematology/Oncology, The Brooklyn Hospital Center, 121 Dekalb Ave, Brooklyn, NY 11201, USA

**Keywords:** Sclerosing epithelioid fibrosarcoma, Skull, Neuraxis

## Abstract

Sclerosing epithelioid fibrosarcoma (SEF) is a rare and aggressive tumor for which no standardized treatment regimens are available. The occurrence of this tumor in children and adolescents has been rarely reported, especially in the head and neck region. Involvement of the neuraxis is reported only in a few patients. We report a case of a 13-year-old boy with SEF of the skull with intracranial extension. The tumor recurred after initial resection and rapidly spread to the brain parenchyma and the meninges with no response to surgery, chemotherapy and radiation therapy.

## Introduction

Sclerosing epithelioid fibrosarcoma (SEF) is a rare tumor which represents a clinically challenging entity. There are no standardized treatment regimens available to treat this tumor [1]. SEF is a clinically aggressive malignancy with a high frequency of local recurrence and distant metastasis [2]. This is a tumor often seen in the adults and occurrence in pediatric age group is rare. A recent systematic analysis shows mean age at presentation of 47 years [3]. Only 10% of patients are younger than 20 years at the time of diagnosis. SEF occurs mainly in the extremities and trunk with relatively rare occurrence in the head and neck region. SEF involving the neuraxis is reported in very few children [2]. We report a case of a 13-year-old boy with SEF of the skull with intracranial extension.

## Case Report

A 13-year-old boy presented to the Pediatric Hematology/Oncology Clinic for evaluation of a lump on the right side of the skull, which was present for more than one year. The swelling was painful to touch with gradual increase in size. There was no history of trauma, fever, headache, vomiting, weight loss, visual disturbances or local redness. Patient was born after an uncomplicated pregnancy and delivery. His perinatal and past medical histories were unremarkable. There was no significant family medical history. He had normal growth and development and was up-to-date with his immunizations.

On physical examination, patient was well nourished and his vital signs were normal. Examination of the head revealed a swelling measuring 3 х 3 cm on the right parietal bone, hard in consistency, tender to palpation, with no other signs of inflammation. Examination of the ears, eyes, nose and throat showed no abnormalities. No lymph nodes were palpable in the neck, axillae or groins. Neurologic examination was normal. Rest of the physical examination did not reveal any abnormality.

**Figure 1:**
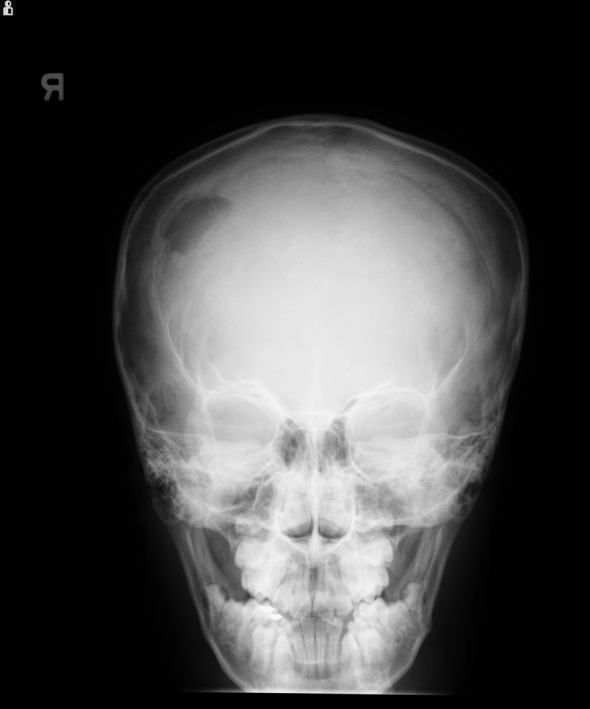
X-ray of the skull showing lytic lesion in the right parietal region.

Laboratory results showed: hemoglobin - 12.4 g/dL, white blood cell count - 6,900/µL, and platelet count - 281,000/µL. Skull X-ray revealed a lytic lesion of the right parietal bone (Fig. 1). Computerized tomography (CT) scan of the head demonstrated 2.5 cm destructive lytic lesion at the right parietal bone with soft tissue mass measuring 2.5 х 1.5 cm, extending to the extra-axial intracranial space. Initial diagnosis was suspicious for Langerhans cell histiocytosis. Bone scan and chest CT scan did not reveal any distant metastasis. Patient had resection of the lesion which was reported as grade 1 SEF. Post-operative head CT scan and MRI showed residual lesion of 8 х 6 mm at the inferior and posterior aspect of the previously excised mass. Considering this rare tumor's poor response to chemotherapy and radiation, a decision was made to do a total resection. A right parietal craniotomy with gross total resection of the tumor, followed by cranioplasty was performed. Pathology report was consistent with SEF as before.

**Figure 2:**
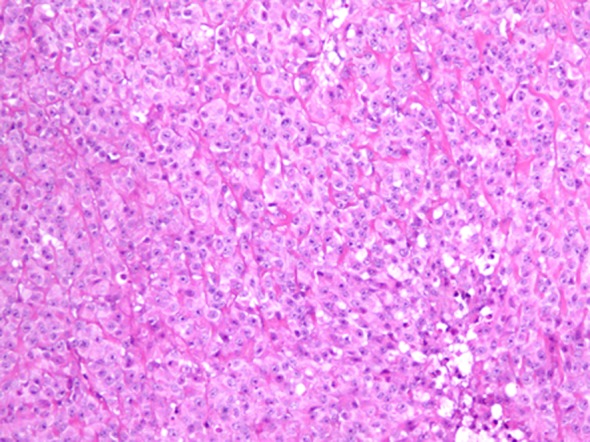
Light microscopic picture of SEF showing tumor cells arranged in hyalinized stroma (hematoxylin and eosin х 10).

Follow-up CT scan and MRI done after 6 months were negative for tumor recurrence. Ten months after the initial presentation, patient presented with an enlarging lump over the surgical site with headache, vomiting and 12 pounds weight loss. MRI of the brain revealed three new enhancing soft tissue lesions at the original tumor site and one at the deep posterior margin of the previous surgical cavity. Positron emission tomography (PET) scan did not show any evidence of distant metastasis. Patient underwent gross total resection of the tumor. Pathology confirmed the diagnosis of grade 3 SEF, more aggressive than the initial presentation (Fig. 2). Post-operative course was uneventful and MRI of the brain demonstrated no residual lesion. Patient was started on chemotherapy with Doxorubicin and Ifosfamide with Mesna. He also underwent external beam cranial irradiation and completed 6 cycles of chemotherapy. At the end of his last cycle of chemotherapy and 19 months after the initial diagnosis, he presented with headache, vomiting, back pain and weight loss. MRI showed cranial and spinal metastasis. There were new enhancing lesions in the right frontal lobe and tumor infiltration of the epidural and subarachnoid spaces from thoracic to sacral areas. Patient's clinical status deteriorated with obstructive hydrocephalus and paraplegia and he died within a month.

## Discussion

Originally described by Meis-Kindblom et al in 1995 as a low grade fibrosarcoma [4], SEF is a slow growing malignancy known for its local recurrence and distant metastasis. Incidence of this tumor is rare with only about a hundred cases reported in the literature so far. Occurrence in children is extremely rare with inconsistent response to therapy. Only 10% of patients are younger than 20 years at the time of diagnosis. SEFs mainly present as tumors of the lower extremities/limb girdle (39%) and trunk (21%) followed by upper extremities (14.5%), the head and neck (14.5%) and abdominal/inguinal areas (11%) [3].

Our patient was diagnosed one year after noticing a slow growing lump in the skull. A recent meta-analysis showed that the mean duration from onset of symptoms to diagnosis was 33 months (range 1 month - 13 years) [3]. The median interval to local recurrence was 4.8 years (range 2.3 - 11 years) and to metastasis 7.7 years (range 4.7 - 14 years) [2]. Up to 27% of patients had distant metastases at the time of diagnosis. Our patient had local recurrence after 10 months and distant metastasis after 19 months of diagnosis. Central nervous system involvement has poor prognosis as evident from our patient. Previous reports also show unexpectedly aggressive clinical behavior of SEF involving nervous system, compared with those arising in more typical locations [2].

Imaging and histopathology are the primary modes of diagnosis. Bone involvement presents as lytic lesions in X-ray. CT scan or MRI results depend on the site of the tumor. Typical histology of SEF consists of epithelioid cells arranged in strands, nests, and/or sheets, in a fibrotic and hyalinized stroma [5]. Mitotic figures are either scant or absent, and necrosis is uncommon. Although histomorphology of SEF suggests the tumor to be low grade, it could manifest signs of high-grade tumor clinically. SEF belongs to the family of fibrosing fibrosarcomas and appears to be the most malignant variant of this family of low-grade fibrosarcomas [6, 7]. The only immunostaining marker consistently reported positive, as in our patient, is Vimentin which is a general marker for soft tissue sarcomas and therefore not specific for SEF [3].

Due to the rarity of this tumor, there are no established treatment regimens. Patients have been treated with amputation, wide excision, radiation and chemotherapy, or various combinations thereof [3]. We treated the patient with surgery, radiotherapy and chemotherapy using Doxorubicin and Ifosfamide. Multiple chemotherapeutic regimens have been used in the previously reported cases with varying results [1, 2].

In conclusion, SEF is a rare and aggressive tumor in children with no specific curative treatment strategies. Diagnosis of SEF needs to be considered in children with unexplained lytic lesions of the bone.
